# Mitochondrial ATP synthase inhibition and nitric oxide are involved in muscle weakness that occurs in acute exposure of rats to monocrotophos

**DOI:** 10.1080/15376510802455354

**Published:** 2009-06-30

**Authors:** S. Venkatesh, A. Ramachandran, A. Zachariah, A. Oommen

**Affiliations:** 1 Neurochemistry Laboratory, Department of Neurological Sciences, Christian Medical College, Vellore, India; 2 Wellcome Trust Research Laboratory, Christian Medical College, Vellore, India; 3 Medicine Unit I & Infectious Disease, Christian Medical College, Vellore India

**Keywords:** ATP synthase, Electron transport chain, Monocrotophos, Nitric oxide, Organophosphate poisoning, Skeletal muscle

## Abstract

Organophosphate poisoning in the context of self-harm is a common medical emergency in Asia. Prolonged muscle weakness is an important but poorly understood cause of morbidity and mortality of the poisoning. This study examined mitochondrial function and its modulation by nitric oxide in muscle weakness of rats exposed to an acute, oral (0.8LD_50_) dose of monocrotophos. Muscle mitochondrial ATP synthase activity was inhibited in the rat in acute exposure to monocrotophos while respiration per se was not affected. This was accompanied by decreased mitochondrial uptake of calcium and increased levels of nitric oxide. Reactive cysteine groups of ATP synthase subunits were reduced in number, which may contribute to decreased enzyme activity. The decrease in ATP synthase activity and reactive cysteine groups of ATP synthase subunits was prevented by treatment of animals with the nitric oxide synthase inhibitor, L-N^G^ Nitroarginine methyl ester, at 12 mg/kg body weight for 9 days in drinking water, prior to monocrotophos exposure. This indicated a role for nitric oxide in the process. The alterations in mitochondrial calcium uptake may influence cytosolic calcium levels and contribute to muscle weakness of acute organophosphate exposure.

## Introduction

Organophosphorous pesticide poisoning is a common method of intentional self-harm in agricultural communities across Asia (Eddleston and Phillips 2004). Organophosphates inhibit acetylcholinsterase (AChE), leading to an accumulation of acetylcholine at cholinergic synapses in the central nervous system and at neuromuscular junctions, and that results in overstimulation of these systems. In severe acute organophosphate poisoning (OPP) this can result in an autonomic cholinergic storm and lead to prolonged neuromuscular weakness and paralysis. The pathophysiology underlying muscle weakness that occurs in acute OPP is not completely clear, and thus there is no specific treatment for it. Consequently it is a major cause of morbidity and mortality of hospitalized OPP patients (Samuel et al. 1995).

Severe, persistent inhibition of AChE is the primary event responsible for muscle weakness that occurs in OPP. However it is unlikely to be the only factor, as cholinergic symptoms are often absent in prolonged muscle weakness and muscle power recovers despite persistent cholinesterase inhibition.

Functional mitochondria are essential in maintaining the integrity of the high energy requiring skeletal muscle. Muscle weakness often occurs subsequent to dysregulation of mitochondrial respiration and energy production, and consequent perturbation of intracellular Ca^2+^ levels (DiMauro and Gurgel-Giannetti 2005; Sarnat and Marin-Garcia 2005). It is possible that, during acute OPP, inhibition of mitochondrial function in the muscle also contributes to muscle weakness. This was studied in paralyzed skeletal muscle, in the early phase of poisoning, in rats subjected to severe acute monocrotophos exposure, an organophosphorus pesticide commonly used in India for intentional self-harm.

Acetylcholine, acting through its receptor, is known to increase cytosolic Ca^2+^ released from the endoplasmic reticulum, resulting in activation of calmodulin that stimulates nitric oxide synthase (NOS) and production of nitric oxide (NO). Increased levels of NO are a natural sequence to the inhibition of AChE by organophosphates. This study also examined nitric oxide signaling, activated in OPP, in modulating muscle mitochondrial function during the early phase of poisoning, as nitric oxide is known to modify mitochondrial proteins and disrupt mitochondrial function, including the ability to control calcium (Dedkova and Blatter 2005).

## Materials and methods

n-Dodecyl-maltoside, Tricine, aminocaproic acid, Brilliant blue G and R, Cytochrome C (from bovine heart), phosphoenol pyruvate, pyruvate kinase, lactic acid dehydrogenase, oligomycin, sodium succinate dibasic hexahydrate, glutamate (disodium salt), rotenone, Nagarse (proteinase enzyme III), biotin-maleimide, and L-N^G^ Nitroarginine methyl ester (L-NAME) were obtained from Sigma Aldrich Inc. (St Loius, MO). Streptavidin linked secondary antibodies and streptavidin peroxidase were from Bangalore Genei Pvt, Ltd. (Bangalore, India). Mouse monoclonal antibodies to α, β, and oligomycin sensitivity conferring protein (OSCP) subunits of ATP synthase were from Invitrogen (USA). Bovine serum albumin was from National Biochemical Corp. (USA). Monocrotophos (36% SL), containing no other compounds, was from Syngenta India Pvt Ltd. (India). All other chemicals were of the highest purity available.

### Animal studies

Wistar rats (female, 150 ± 5 g) were administered monocrotophos by gavage at a dose of 0.8 LD_50_ in 200 μl water (Monocrotophos LD_50_ = 18 mg/kg body weight) (Manhande and Chopde 1995). Control rats were administered 200 μl water by gavage. Animals were evaluated after exposure to monocrotophos for cholinergic signs (chewing, tremor, salivation, lacrimation, and diarrhea) and for muscle power (Grade 0: normal mobility, Grade 1: ataxic gait, Grade 2: stretch movements after tail stimulation or Grade 3: no voluntary movements after tail stimulation) (De Bleecker et al. 1991). Monocrotophos was administered between 8 and 9 am on days of study and animals were sacrificed 2.5 h later when they were paralyzed (no voluntary movements after tail stimulation). Animals were euthanized under ether anesthesia. Blood (2.0 ml) was collected from each rat on sacrifice and RBC and plasma separated and stored at −70°C until assay. For inhibition of nitric oxide synthase (NOS), L-NAME (hydrochloride) was given to rats in drinking water at a dose of 0.1 mg/ml/day (12 mg/kg body weight/day) for 9 days (Thomas et al. 2001), following which they were administered monocrotophos as mentioned above. Each parameter was studied in a minimum of six rats. The study was approved by the Institutional Review Board and Institutional Animal Ethics Committee of the Christian Medical College, Vellore.

### Skeletal muscle mitochondrial isolation

On sacrifice, upper and lower limb skeletal muscles were immediately dissected from the animal and rinsed with 50 mM Tris HCl pH 7.4 containing 100 mM KCl/2 mM EGTA. Five grams of muscle were finely minced and incubated for 3 min in eight volumes of buffer containing Nagarase (2.1 U/ml), 1 mM ATP, 5 mM MgCl_2_, 0.5% BSA, homogenized in a Dounce homogenizer and centrifuged at 490 g for 10 min at 4°C to obtain the post-nuclear supernatant. This was filtered through nylon cloth and the filtrate spun at 10,300 g for 10 min at 4°C. The mitochondrial pellet obtained was washed by re-suspension in 50 mM Tris HCl pH 7.4/100 mM KCl (30 ml) and centrifugation at 10,300 g for 10 min at 4°C. Washing was done twice and the final mitochondrial pellet was suspended in 1.0 ml of buffer. Enrichment of the mitochondrial fraction was assessed by measuring the marker enzyme succinate dehydrogenase (Pennington 1961). Mitochondria isolated from muscle of non-poisoned rats served as controls.

### Assay of cholinesterases

Plasma butyrylcholinesterase and RBC and brain acetyl-cholinesterase were assayed by the method of Ellman et al. (1961) using butyrylthiocholine iodide or acetylthiocholine iodide, respectively, as substrates. Enzyme units were calculated from the molar extinction co-efficient of 5-mercapto–2-nitro benzoic acid of 13.6 × 10^3^.

Protein was estimated by the method of Lowry et al. (1951) using bovine serum albumin as standard.

### Mitochondrial respiration, swelling, and calcium flux

Oxygen uptake was determined polarographically using a Clark type electrode. Mitochondria (300 μg protein) were suspended in 2.0 ml 150 mM sucrose, 1 mM KH_2_ PO_4_, 5 mM MgCl_2_, 20 mM KCl, 10 mM Tris HCl pH 7.4 containing either 5 mM succinate or 5 mM glutamate as respiratory substrates, allowed to stabilize for 3 min and oxygen uptake measured (State 4 respiration). Oxygen uptake stimulated by addition of 0.3 mM ADP was assessed as State 3 respiration. The respiratory control ratio was calculated as the ratio of State 3/State 4 respiration (Madesh et al. 1999). Mitochondrial swelling was assessed by following the drop in absorbance at A540 nm as described earlier (Takeyama et al. 1993), and calcium flux measurements were carried out by quantifying changes in the absorption spectrum of Arsenazo III at 675/685 nm (Scarpa 1979).

### Assay of mitochondrial complex IV and V

Complex IV activity was measured by the oxidation of cytochrome c at 550 nm (Darley-Usmar 1987). Data is represented as the pseudo first order rate constant (*k*) divided by the protein concentration. Complex V was assayed by an ATPase coupled reaction measuring oxidation of NADH at 340 nm at 30°C in the presence and absence of oligomycin (Soper and Pedersen 1979). Units were defined as μmoles NADH oxidized/min/mg protein.

### 2D Blue native gel electrophoresis of mitochondrial complexes

Blue Native polyacrylamide gel electrophoresis (BN-PAGE) was performed using the method developed by Schagger and von Jagow (1991) and Schagger et al. (1994), and modified by Brookes et al. (2002). Mitochondrial pellets (0.5 mg of protein) were resuspended in 80 μl of extraction buffer (0.75 M aminocaproic acid, 50 mM BisTris, and 1% *n*-dodecyl-ß-D-maltoside), incubated on ice for 60 min, centrifuged at 14,000 rpm for 5 min, and the supernatants containing the extracted mitochondrial complexes collected and kept on ice; 2.5 μl of Coomassie Brilliant Blue G-250 (5% w/v suspension in 0.5 M aminocaproic acid) was added to 75 μg of mitochondrial protein and samples stored on ice for no more than 30 min before being subjected to non-denaturing PAGE (5–12% gels) to separate the individual respiratory complexes intact. The identity of the ATP synthase band in mitochondria subjected to BN-PAGE was confirmed by electro-transfer of the gel to PVDF membranes and probing the blots with antibodies to the α-subunit of complex V.

### SDS-PAGE, Immunoblotting, and sulfhydryl group labeling of ATP synthase

In further experiments, the band corresponding to ATP synthase in BN-PAGE was excised from the gel and subjected to 2^nd^ dimension SDS-PAGE (10–14% gels) with β-mercaptoethanol to separate individual proteins in the complex. Control and monocrotophos treated samples were run in duplicate so that one gel could be stained with Brilliant blue R for protein visualization and the other transferred to PVDF membranes and probed with antibodies to ATP synthase subunits α, β, and OSCP or analyzed for free sulfhydryl groups. SDS-PAGE and immunoblotting were performed according to standard protocols (Laemmli 1970; Towbin et al. 1979).

For labeling of free sulfhydryl groups on subunits of ATP synthase, the blots were incubated with biotin-maleimide (12 μg/ml) for 12 h at 4°C, blocked with 2% BSA in PBS Tween 20 (0.02%) for 1 h and incubated with streptavidin-peroxidase (1:5000 diluted) for 45 min. Bands were developed with 0.024% H_2_O_2_/3.2 mM diaminobenzidine for 5 min and quantified using densitometric software developed in-house that correlated with Bio-Rad Quantity One Quantitation software version 4 (r = 0.8). Under these conditions, modification of thiol groups is reflected in decreased labeling relative to control.

### Estimation of nitrate in mitochondria and plasma

Nitrate in mitochondria (100 μg) and plasma (100 μl) was first reduced to nitrite with copper-cadmium alloy and then assayed by the Griess reaction (Sastry et al. 2002).

### Statistical analysis

Parameters under study in monocrotophos treated animals were statistically analyzed for differences from controls by the Mann Whitney U-test using the statistical software package SPSS 11.00. A test was considered statistically significant at *p* < 0.05.

## Results

### Monocrotophos Treatment of Rats

In rats administered monocrotophos (0.8LD_50_) RBC AChE was inhibited 97% and plasma BuChE 91.5% within 2.5 h of poisoning. RBC AChE levels in control rats were 8.96 ± 0.456 units/mg protein that decreased to 0.253 ± 0.218 units/mg protein in monocrotophos treated animals (mean ± SD, n = 6). The values for plasma BuChE levels were 313.23 ± 34.72 units/liter and 26.69 ± 8.98 units/liter in control and monocrotophos treated animals, respectively.

All rats treated with monocrotophos exhibited cholinergic symptoms and muscle weakness. Cholinergic symptoms began at 12.92 ± 5.58 min and continued until 41 ± 12.21 min after acute monocrotophos poisoning. Muscle weakness developed at 37.5 ± 26.94 min and progressed until 57.5 ± 23.67 min (mean ± SD, n = 6). Muscle paralysis occurred within 2 h of poisoning.

### Skeletal muscle mitochondrial integrity and respiration in monocrotophos exposure

Mitochondrial swelling, respiration rates, respiratory control ratios (RCR) using substrates for complex I and II, and the activity of complex IV were not altered significantly in rats exposed to monocrotophos.

### Enzyme activity of muscle mitochondrial complex V in rats exposed to monocrotophos

**Figure 1:**
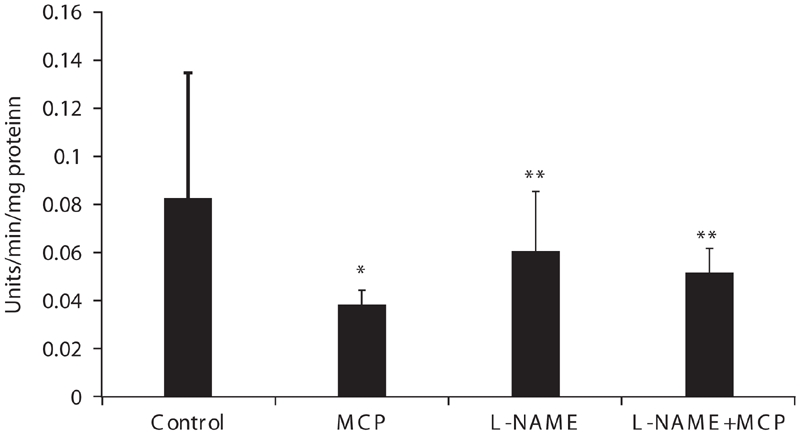
Muscle mitochondrial complex V activity of control, monocrotophos (MCP), L-NAME, and L-NAME + MCP treated rats. Muscle mitochondria were isolated from control, MCP, L-NAME, and L-NAME + MCP treated rats and complex V activity measured. Values are mean ± SD of nine control and six rats in all other groups. * p < 0.05 compared to Control. ** p < 0.05 compared to MCP.

Complex V (ATP synthase) was significantly inhibited by 55% in muscle mitochondria isolated from rats treated with 0.8LD_50_ dose of monocrotophos compared to control animals (Fig. 1).

### Mitochondrial calcium uptake in rats exposed to monocrotophos

**Figure 2:**
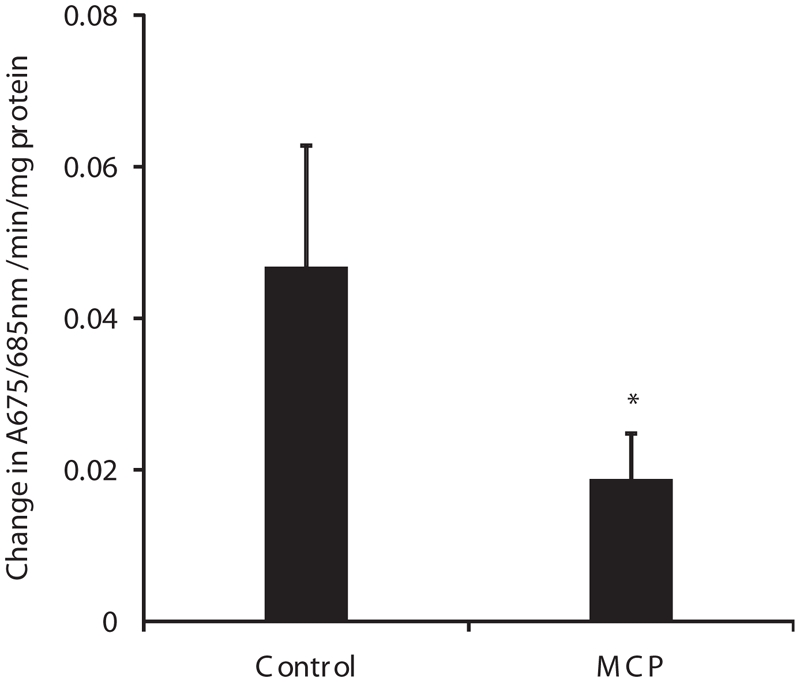
Calcium uptake by muscle mitochondria isolated from control and monocrotophos treated rats. Muscle mitochondria isolated from control animals and after monocrotophos induced paralysis were used for measurement of calcium uptake using the dye Arsenazo III and following absorbance at 675/685 nm. Values are mean ± SD of six rats. *p < 0.05 compared to Control.

The uptake of calcium by muscle mitochondria from monocrotophos treated animals was inhibited 60% compared to control animals (Fig. 2).

### Nitric oxide levels in plasma and mitochondria of rats treated with monocrotophos

**Figure 3:**
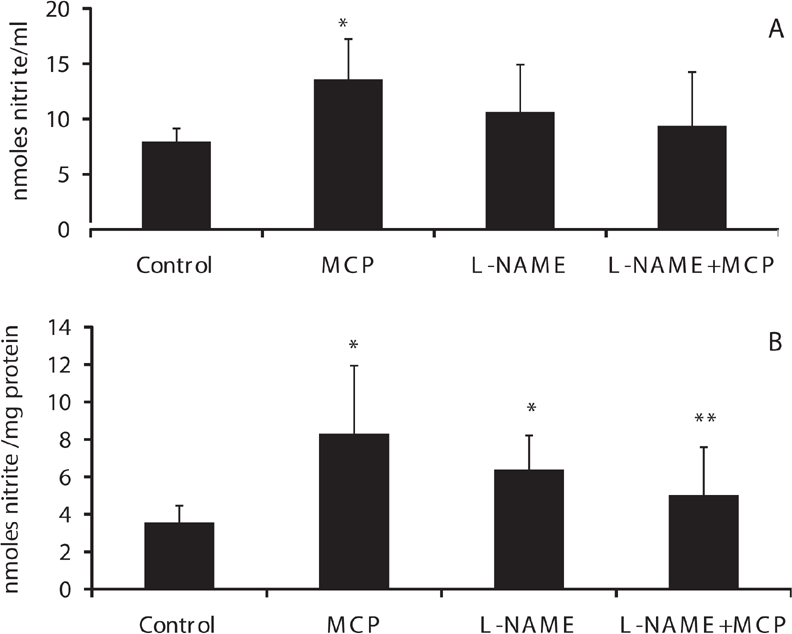
Nitric oxide levels in plasma and muscle mitochondria of control, monocrotophos (MCP), L-NAME, and L-NAME + MCP treated rats. Nitrate, the stable end product of nitric oxide was measured (as nitrite) in plasma (a) and mitochondrial samples (b) from control, MCP treated, L-NAME, and L-NAME + MCP treated animals. Values are mean ± SD of nine control and six rats in all other groups. *p < 0.05 compared to Control. **p < 0.05 compared to MCP.

A significant increase in nitrate (measured as nitrite), the stable end product of NO, was noted in plasma and skeletal muscle mitochondria of paralyzed rats during the early phase of acute monocrotophos exposure (0.8LD_50_ dose) (Fig. 3a and 3b, respectively).

### Modification of reactive cysteines on ATP synthase subunits from rats treated acutely with monocrotophos

**Figure 4:**
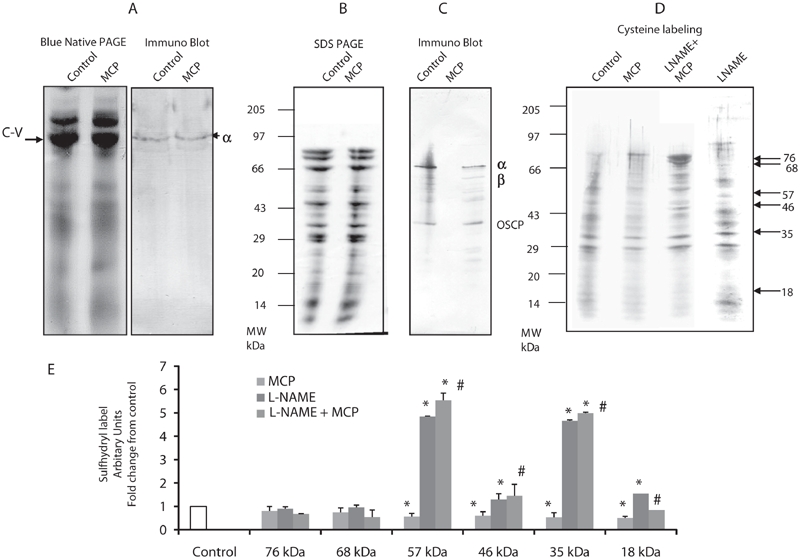
Sulfhydryl group reactivity of muscle mitochondrial ATP synthase from control, monocrotophos (MCP), L-NAME, and L-NAME + MCP treated rats. Muscle mitochondria isolated from control and monocrotophos poisoned rats were subjected to 2D BN-PAGE, immunoblotting and sulfhydryl labeling. (a) First dimension blue native gel after staining with coomassie blue (Complex V (CV) indicated with arrow) and immune blot after probing with the *a*-subunit of ATP synthase. (b) Second dimension SDS-PAGE gel of ATP synthase identified and excised from the 1st dimension gel, stained with coomassie blue. (c) Immune blot of the 2nd dimension SDS-PAGE gel with separated ATP synthase subunits after probing with the anti-α-subunit, anti-*b*-subunit, and anti-OSCP subunit of ATP synthase. (d) Sulfhydryl group labeling on the 2nd dimension SDS-PAGE gel with separated ATP synthase subunits using biotin maleimide in controls and monocrotophos poisoned animals in the presence and absence of L-NAME, an NOS inhibitor. (a–d) are representative of one control and one monocrotophos treated rat. (e) The intensity of sulfhydryl labeled bands in (d) were determined and plotted as fold change from control. Values are mean ± SD of three rats. *p < 0.05 compared to Control. #*p* < 0.05 compared to MCP treated rats.

Protein levels of ATP synthase on 1D BN-PAGE (Fig. 4a) and of ATP synthase subunits in the second dimension SDS PAGE, from gels stained for total protein, did not differ between control and monocrotophos treated rats (Fig. 4b). Blots probed for α, β, and OSCP subunits of ATP synthase resulted in detection of proteins at approximately 58, 52, and 27 kD (Fig. 4c).

Sulfhydryl labeling indicated that a number of ATP synthase subunits were modified in acute monocrotophos exposure. Six subunits with reactive cysteines were labeled under these conditions. The one at 57 kD showed approximately 43% decrease in labeling, while the one at 18 kD showed a 50% reduction in labeling compared to controls (Fig. 4d and 4e). The fact that the molecular weights of these proteins correlated closely with those obtained on the immunoblots for the α, β, and OSCP subunits suggested that these subunits were modified. In addition a 46 kD and 35 kD band also showed 40% and 50% decrease in labeling, respectively. Sulfhydryl group modification appeared to be specific (to certain subunits) and not a universal phenomenon in monocrotophos poisoning, since the two bands at 76 kD and 68 kD did not show any change in labeling after poisoning (Fig. 4d and 4e).

### Inhibition of NOS and ATP synthase activity after monocrotophos exposure

L-NAME, a general NOS inhibitor, treatment of animals increased plasma and muscle mitochondrial levels of nitrate compared to control animals, although the increase was not significant (Fig. 3a and 3b). L-NAME, however, prevented the increase in NO levels seen during monocrotophos exposure in plasma and significantly in muscle mitochondria (*p* < 0.05) (Fig. 3a and 3b, respectively).

Complex V activities (ATP synthase) did not differ significantly between control, L-NAME, and L-NAME + monocrotophos treated animals (Fig. 1). This indicated that L-NAME inhibition of NOS prevented inhibition of ATP synthase induced by monocrotophos exposure, which suggested that NO had a role to play in this process.

Prevention of NO synthesis by L-NAME significantly increased sulfhydryl labeling of ATP synthase subunits 57, 46, 35, 18 kda by 4.5-, 1.3-, 4.7-, and 1.6-fold, respectively, compared to control animals (*p* < 0.05) (Fig. 4d). This significant increase in sulfhydryl label of these ATP synthase subunits was preserved in animals treated with L-NAME prior to monocrotophos treatment compared to those treated only with monocrotophos (*p* < 0.05) (Fig. 4). This data further supports the role of NO in the modification of sulfhydryl groups on ATP synthase in monocrotophos exposure.

## Discussion

OPP results in rapid toxicity due to efficient absorption and high affinity of binding to AChE of these compounds. The nature and rapidity of the toxicity may explain why cellular events that occur in the early phase of poisoning initiate prolonged muscle pathology. Muscle weakness of OPP patients is associated with muscle damage that occurs early in the poisoning (John et al. 2003). This study was to elucidate intracellular mechanisms in muscle that occur soon after acute OPP that may contribute to muscle injury and focused on the mitochondria.

It was found that acute exposure to monocrotophos did not affect skeletal muscle mitochondrial respiration or coupling to oxidative phosphorylation which need to be tightly coupled in order to generate ATP for cellular energy. The activity of ATP synthase was, however, decreased by 55%. This would result in levels of ATP which may be insufficient to maintain the integrity of the cell (Wilkinson and Robinson 1974) and therefore contribute to muscle weakness and injury that occurs in acute OPP.

Mitochondria also contribute to maintaining calcium homeostasis in the muscle by calcium uptake through a uniporter, a process that requires ATP (Moreau et al. 2006). The inhibition of energy-dependent mitochondrial Ca^2+^ influx observed in this study may lead to elevated sarcoplasmic Ca^2+^ that can injure the muscle. Muscle damage induced within 2 h of exposure to organophosphates has been shown earlier by Leonard and Salpeter (1979) to be a myopathy mediated by calcium.

Interventions that prevent or reduce ATP synthase inhibition in OPP may decrease the resulting muscle injury. Increased NO levels are known to disrupt muscle contraction, cause muscle weakness and fatigue, and to underlie muscle degeneration and toxicity of organophosphate poisoned rats (Jeyarasasingam et al. 2000; Gupta et al. 2002; Smith and Reid 2006). The data in this study also showed increased systemic levels of NO in acute monocrotophos exposure that correlated to the inhibition of ATP synthase, as inhibition of NOS and reduction of NO levels resulted in partial recovery of ATP synthase activity. These results support the studies of Gupta et al. (2002) that show a marked decrease in rat muscle and brain ATP levels, within an hour of treatment with the organophosphate compound diisopropylphosphorofluoridate. This decrease was accompanied by an increase in NO levels. Nitric oxide is also implicated in toxicity to the brain in organophosphate pesticide and nerve agent poisoned rats (Chang et al. 2001; Damodaran et al. 2006). In the brain, organophosphates disrupt respiration by inhibition of Complex I/IV (Yen et al. 2004). This clearly differs from the situation in organophosphate poisoned paralyzed muscle where NO targets Complex V (ATP synthase).

NO is known to modify reactive cysteines on proteins through post-translational modification and initiate signaling (Jaffrey et al. 2001). To explore the possibility of such modifications on mitochondrial ATP synthase, we used a proteomics approach coupled to labeling reactive cysteines with biotinylated maleimide. The labeling of four subunits of ATP synthase was substantially decreased in monocrotophos exposure. This suggested that certain subunits of ATP synthase are susceptible to thiol modification subsequent to acute monocrotophos exposure. NO may play a role in this modification as it was prevented by L-NAME treatment.

L-NAME treatment was expected to reduce NO levels to less than those in controls. Paradoxically, plasma and mitochondrial NO levels were higher, although not significantly, in the L-NAME treated animals compared to controls. Chronic low dose L-NAME treatment of rats is known to activate NO synthesis through negative feedback regulation of NOS by NO and may also account for the observations in this study (Bernatova et al. 2007). The lower NO levels in L-NAME + monocrotophos treated animals compared to those treated only with L-NAME lend support to negative feedback regulation of NOS by NO in animals on the treatment schedule of this study.

L-NAME inhibition of NO synthesis is associated with increased levels of glutathione (GSH) in animals (Schild et al. 2003). Under these conditions NO induced by negative feedback inhibition of NOS may be rapidly oxidized by GSH to GSNO, the transport and storage form of NO. This sequestration of NO may explain the increased free sulfyhdryl groups on ATP synthase subunits observed in L-NAME and L-NAME + monocrotophos treated rats compared to control animals during the initial phase of poisoning.

In conclusion, the studies demonstrate that muscle mitochondrial ATP synthase is inhibited in paralyzed muscle during the early phase of acute monocrotophos exposure, an effect possibly mediated through NO. The inhibition of ATP synthase may be responsible for reduced calcium uptake by the mitochondria, which can affect cellular calcium homeostasis and lead to muscle damage. As inhibition of NOS appears to protect against modification and inhibition of ATP synthase, scavenging NO early in OPP may help maintain myocyte integrity and prevent muscle weakness. This is currently under study.

## Abbreviations

OPP: organophosphate poisoning
MCP: Monocrotophos
AChE: acetylcholinesterase
BuChE: Butyrylcholinesterase
L-NAME: L-N^G^ Nitroarginine methyl ester
RBC: Red blood cells
BN-PAGE: Blue Native Polyacrylamide Gel Electrophoresis
OSCP: Oligomycin sensitivity conferring protein
NOS: Nitric oxide synthase
NO: Nitric Oxide
